# Infectious Diseases and Vaccination for Rabies

**Published:** 1884-10

**Authors:** M. Pasteur


### Selections.

### INFECTIOUS DISEASES AND VACCINATION FOR RABIES.

*The Medical Times and Gazette* (August 23, 1884) publishes the following version of M. Pasteur's address, delivered at the International Medical Congress at Copenhagen, August 11, 1884:

GENTLEMEN: In addition to the fact that they are meetings at which the most important problems of medicine are subjected to examination, congresses also serve to indicate to posterity the chief directions of progress. Three years ago, when the congress met in London, the microbe theory in its application to the ætiology of infectious diseases, was still the subject of violent attack. Many who opposed themselves to advanced ideas persisted in maintaining that diseases exist "in us, from us, and by us."

One would imagine that those who hold that these diseases arise spontaneously would in London have shown themselves eager to defend this thesis, but opponents to the doctrine that the principal cause of infectious diseases is an external one did not venture to come forward, and discussion on this question was not even opened. In such an assembly as this, when all are prepared for a new triumph of truth, we see at length a way open for these men to give in to it.

For the rest, all clear-sighted men had foreseen that, as soon as one would be able to show that the spontaneous origin of the microscopic life was a chimerical hypothesis, and, on the other hand, that this microscopic life had relations to organic decomposition and fermentation, theories as to the spontaneous origin of disease would cease to exist. In like manner it is from the London Congress that another important advance dates its confirmation, namely, the possibility of attenuating the different viruses, varying their infectivity, and preserving them by means of suitable cultures, and lastly, the application of this discovery to veterinary medicine. To the vaccine microbes of fowl cholera and splenic fever, one has been able to add others; and the animals that are protected against the attacks of fatal infectious diseases are now to be counted by hundreds of thousands. The violent opposition which this innovation has encountered will soon be swept away in the current of new ideas. Is the application of this new advance, then, to be confined in the future to the prevention of the diseases of animals? Besides the fact that one need never despair of a discovery and its fruitfulness, can we say that this question is already solved in its principal points? Splenic fever, for example, is common to animals and man; and we can say for certain that, if it were worth while, nothing would be simpler than to produce in man insusceptibility to this disease. The procedure which is successful in animals would be applicable without modification, so to speak. It would simply depend on one's proceeding with an extraordinary degree of caution, such as the life of an ox or a sheep does not require. Instead of vaccinating with vaccine of only the second degree, one could take three or four of variable virulence, till one chose the first, weak enough not to cause the slightest symptom, but which would yet produce insusceptibility to the disease. In the case of human diseases the difficulties lie then, not in the application of the new prophylactic method, but rather in the knowledge of the physiological properties of the virus. To attenuate the virus to the proper degree, it is necessary to control our efforts by experiment; but the experiments which are allowable in animals are criminal when we have to do with man. In relation to the diseases which exclusively affect mankind, this is the chief cause of the difficulties of the investigation. Let me in the mean time remind you that the inquiry of which we are speaking dates, so to speak, from yesterday, that it has already been fruitful in results, and that we have a right to expect new advances when we obtain a closer acquaintance with the diseases of animals, especially those which attack both man and beast. It was the desire to penetrate further into this two-fold knowledge which induced me to study rabies, in spite of the obscurity in which that disease was enveloped.

It is now four years since the study of rabies was first commenced in my laboratory, and it has been continued without any other interruption than the enforced cessations which depend on the conditions of the inquiry—conditions which are very unfavorable. The incubation of the disease is always of long duration. There are never sufficient facilities to enable one at a given moment to multiply experiments. In spite of these material hindrances, which, however, the French Government, in its care for the great scientific interests involved, has done everything in its power to remove, the experiments which we, my fellow-workers and I, have carried out, have, nevertheless, passed beyond the possibility of numbering them. To-day, gentlemen, I shall only describe the most recent results of our inquiries. Every disease, and especially such a disease as rabies, immediately makes one think of its cure, but to set one's self forthwith to search for remedies is to expose one's self to what is only too often a fruitless labor. It is in a manner to trust to accident for advance. Better is it to undertake in the first place to study the nature of the disease, its cause and development, in the distant hope of thereby discovering means of preventing it. If the problem of rabies is to-day no longer insoluble, it is to these last-named methods that we owe this advance. Thus we have proved that the virus of rabies always develops itself in the nervous system, in the brain, the spinal cord, the nerves, and the salivary glands, and never simultaneously invades every part. It may, for example, fix itself in the spinal cord, and then attack the brain; or one may find it in one or more parts of the brain and not in others.

If one kills an animal when the disease is at its height, it is often difficult to find the virus of rabies at any given point in the brain or spinal cord, but we have fortunately discovered that, in every case in which death occurs as a natural result of the development of the disease, the uppermost portion of the spinal cord, which forms the point of transition between the cord and the brain, and which one calls *the bulb*, is invariably the seat of the poison. When an animal dies of rabies (and we know that the disease invariably ends in death) it is absolutely certain that one will be able to obtain from the animal's *bulb* rabies virus, which will produce the disease by inoculation on the surface of the brain in the arachnoid cavity, after previous trephining.

If you take any street-dog you please and inoculate rabies in this manner by trephining, using as inoculating material a portion of the bulb of an animal which has died of the disease, you will invariably convey rabies. The dogs to which the disease has been communicated in this manner are to be counted by hundreds. The method has never failed. The same operation has been performed on hundreds of Guinea pigs and on a yet greater number of rabbits, without a single failure.

These two remarkable results—the invariable presence of the virus in the bulb of animals dead of the disease, and the certainty that one can communicate rabies by inoculation in the arachnoi cavity—are axioms firmly established by experiment, and are of extreme importance. Thanks to the careful application and the so to speak daily employment of these criteria, we were able to proceed with certainty in a study of such difficulty. But, however solid this experimental basis may be, it is not in itself able to show us the way to a vaccination method against rabies. In the present position of science, to presuppose the discovery of a means of preventing infectious disease by vaccination—(1) one must have at one's disposal a virus which can exist in different degrees of virulence, the weakest of which may serve as a vaccine; (2) one must have discovered a method of producing these varying degrees of virulence. Hitherto science has known only one kind of rabies, that which occurs in dogs. All hydrophobia, in dogs, men, horses, cattle, wolves, foxes, etc., comes originally from the bite of a mad dog. Rabies never arises spontaneously, either in the dog or any other animal.

None of the instances on record of rabies occurring spontaneously are really authentic; I will add that in making this assertion I do not ignore the fact that there must have been a *first* case of rabies. To come forward with this kind of objection when it is a question of solving the inquiry which engages us, helps no one, but touches a problem which even now is still inscrutable—the very problem of life. It is like answering one who should maintain that an ovum always originates from an ovum, but, nevertheless, that the first ovum must have originated spontaneously. Science, which knows itself, sees that it benefits nobody to argue about the origin of things; it sees that such origin for the moment at least lies beyond its province.

In short, the inquiry whether the rabies virus can occur in different degrees of virulence, like the virus of fowl cholera, of splenic fever, etc., is the first question to solve in order to arrive at the prophylaxis of hydrophobia. But how does one obtain the knowledge that there are various possible degrees of virulence in the virus of fowl cholera? And to what signs does one have recourse to determine the strength of a virus which kills whenever it infects?

Does rabies present any symptoms to help us? No, these symptoms are very variable. They depend essentially on the brain or spinal cord, where the virus instantly concentrates itself and flourishes. The mildest rabies that occurs—for such does occur—may in another animal of the same species produce the most violent rabies. Can one then determine intensity by means of the duration of the incubation period? No, nothing is more variable. A mad dog bites several dogs; one of these goes mad after an interval of a month or six weeks, another after two or three months' interval, and so on. Further, nothing can be more variable than the duration of the incubation period, according to the various modes of inoculation. Does one never see hydrophobia now occur and now fail to occur after a bite or hypodermic injection under exactly similar circumstances in every other respect, while inoculation on the surface of the brain never fails, and the incubation period is in such case of proportionally short duration?

Nevertheless, it is possible to determine with sufficient certainty the strength of a rabies virus according to the duration of the incubation, but on two conditions : one must make use of intracranial inoculation, and one must bear in mind that the manner in which inoculation takes place furnishes one of the most powerful sources of irregularity in the results, according as inoculation is by bite, by hypodermic or intravenous injection. The duration of incubation may really depend very much on the quantity of active virus, that is, the virus which reaches the nervous system without diminution or change. Notwithstanding that the quantity of virus which will produce rabies may be, so to say, infinitely small—it has been shown that, as a general rule, hydrophobia occurs in consequence of a bite, whereby the quantity of virus introduced into the body must generally be so small as to be almost indefinable—it is easy to double the length of the incubation, simply by taking a still smaller proportion of the small quantity inoculated. I will quote some examples:

On May 10th, 1882, there were introduced into the popliteal vein of a dog ten drops of a fluid which had been obtained by macerating in three or four times its weight of sterilized broth a portion of the bulb from a dog which had died of rabies after being found in the streets in a mad condition.

A second dog was inoculated with a hundredth part of the quantity, and a third dog with a two hundredth. The first dog was seized with rabies after an incubation period of eighteen days, the second after thirty-five days, the third remained unaffected, i. e., in this last case, and by the method of inoculation used in this experiment, a certain quantity of virus proved insufficient to produce rabies. This last dog was susceptible of rabies, as all dogs usually are, for it was again inoculated on September 3, 1882, and was seized with rabies twenty-two days later.

I take another example, which occurred in rabbits, and in which another mode of inoculation, viz., trephining, was employed: The bulb of a rabbit, which had died of rabies after inoculation with a very virulent virus, was dissolved in two or three times its bulk of sterilized broth. After it had been allowed to stand for some few seconds, two drops of the supernatant liquid were inoculated into a rabbit by trephining, another was inoculated with a fourth of that quantity, and other rabbits were subsequently inoculated with $\frac{1}{16}$ $\frac{1}{64}$, $\frac{1}{128}$, and $\frac{1}{125}$ of the same quantity respectively. All these rabbits died of rabies, and the length of incubation was eight days in the first, nine in the second, ten in the third and fourth, and twelve to sixteen days in the two last. This variation in the period of incubation was not occasioned by an attenuation of the actual virulence of the virus, such as might be produced by solution, for there was a return to the eight days' incubation period if fresh rabbits were inoculated with the virus obtained from these rabbits after death. We see from these examples that, in the case where rabies is produced by a bite or by hypodermic injection, interference with the length of the incubation period must be chiefly ascribed to the great variation which is possible in the amount, always indefinite, of inoculated poison which reaches the central nervous system.

If, then, we wish to determine the intensity of the virus from the length of the incubation period, it is unavoidably necessary to have recourse to inoculation by trephining, which is absolutely certain in its effects, and to employ larger quantities than such as would be necessary simply to produce rabies. When we operate in this way, irregularities in the length of incubation with the same virus will show a tendency to entirely disappear, because we always obtain the maximum of effect which the virus can produce, that maximum corresponding to the minimum duration of incubation.

Thus we have at length obtained a method which has enabled us to inquire into the possible existence of varying degrees of virulence, and to mutually compare them. The only secrets in this method, I repeat, are to inoculate by trephining, and to use a quantity of virus which, although very weak, is more than sufficient to produce rabies in and by itself. This method eliminates the causes which might interfere with the duration of the incubation period, and makes it dependent exclusively on the activity of the virus, the comparative strength of which varies according to the minimum duration of incubation, which is determined by its effect.

The first application of this method was in connection with the study of rabies, and expressly in connection with attempts to discover if rabies is always one and the same, only with such differences as the varying nature of different kinds of dogs might produce. Let us, then, take wandering mad dogs at any season you please in the same year, or in different years, and belonging to the most different varieties. Let us in each case isolate the bulb, and with the material obtained from it inoculate, by trephining, from one to two rabbits, using two drops of the fluid obtained by macerating the bulb in two or three times its bulk of sterilized liquid—all proper cleanliness being observed. Let the inoculation be performed with the help of a Pravaz's syringe, somewhat bent at the end, introducing it through the dura mater into the arachnoid space. The following will be observed: In all the rabbits, no matter what mad dog was used to inoculate them from, the incubation period will fall almost without exception between the twelfth and fifteenth days - you will never meet with an incubation period of eleven, ten, nine, or eight days, though you may sometimes meet with periods of several weeks or several months.

Rabies thus, clearly enough, possesses one poison only: its modifications, which, however, are very limited, depend simply on the known difference in the susceptibility of various kinds of dogs. But we shall now see a very marked change in the virulence of rabies virus.

Let us take after death one of the numerous rabbits which we have inoculated with the virus taken from a mad dog, and let us introduce two drops of its bulb, prepared in the way given above, into another rabbit, whose bulb again shall serve to inoculate a third rabbit, and its bulb again to inoculate a fourth, and so on. You will then observe, even from the first transmission, a diminution in the incubation period in the various rabbits. I will give an example:

In the last month of 1882, fifteen cows and one bullock died of rabies on one farm near Melun, a principal town in the Department Seine et Marne, in consequence of having been bitten on October 2d by the farm dog, which had gone mad. The head of one of the cows, which died on November 19th, was sent to the laboratory of M. Rossignol, a veterinary surgeon at Melun. Numerous experiments, performed on dogs and rabbits, showed that only the following parts were the seat of the virus, viz: the cerebrum, bulb, and cerebellum, the frontal and temporal lobes. The rabbits which were inoculated from these parts were seized with the disease on the seventeenth or eighteenth day after inoculation. With the bulb taken from one of these rabbits after death two other rabbits were inoculated, one of which was attacked by the disease on the fifteenth day, and the other on the twenty-third day after inoculation. I will observe once for all that, if we transmit rabies from one animal to another of a different species before the virus has reached its maximum in the former, considerable irregularity is met with in the duration of the incubation period. We have here an instance of this, for the same virus in one rabbit gave an incubation period of fifteen days, and in the other one, of twenty-three days, though all the other conditions were apparently similar.

With the bulb taken from the former of these two dead rabbits, two other rabbits were inoculated. In one of these the disease appeared after an interval of ten days; in the other after an interval of fourteen days. The bulb of the former of these was in like manner used to inoculate two more rabbits, in whom the disease appeared after intervals of ten and twelve days respectively. In the fifth transmission to two rabbits, the disease appeared in both after eleven days' interval; in the same manner in the sixth transmission the disease appeared after eleven days' interval, in the seventh after twelve days' interval, in the eighth after ten days' interval, in the ninth also after ten days' interval, in the tenth after nine days, in the eleventh after eight days, in the twelfth after nine days, and so on, with variations of not more than twenty-four hours at the outside, right down to the twenty-first transmission, when rabies appeared after eight days' interval; the same interval of eight days was obtained in further transmissions, down to the fifteenth, which has recently taken place. This series of experiments, which commenced on the 19th November, 1882, is still in progress.

Allow me at this point to draw your attention to the extreme certainty and facility which characterize trephining and subsequent inoculation with rabies virus; for on every twelfth day for a period of about twenty months, a succession of rabbits have been trephined and inoculated with a rabies virus procured from a single individual, and that without any interruption in the success of the experiments.

Guinea-pigs most rapidly attain the maximum virulence peculiar to them. In these animals the incubation period, which varies and is irregular at the beginning of successive transmissions, quickly attains a definite duration of five days. Seven or eight transmissions from Guinea-pig to Guinea-pig brings us to the maximum virulence. Moreover, both in Guinea-pigs and rabbits, one observes, according to the origin of the virus, variations in the number of transmissions required to obtain the maximum virulence. If we transmit this maximum degree in rabbits and Guinea-pigs to dogs, we obtain a virus which far surpasses in virulence that of the rabies which is commonly met with.

But I hasten to say that, whatever may be the usefulness of the discovery which I have just described, there exists and can be produced different kinds of rabies—all of which are more violent and kill more rapidly than the rabies which occurs in dogs. Scientific men overlook nothing which can be discovered in the field of science, but many whom the very thought of rabies strike with fear look for something more than scientific curiosities. How much greater interest would man have in becoming acquainted with a rabies virus which had been attenuated in its virulence! One, then, might cherish a hope of obtaining a vaccine from the rabies virus such as we have obtained in fowl cholera, splenic fever, and even acute septicæmia. Unfortunately, the methods of procedure which were used in regard to these poisons proved themselves inapplicable in dealing with the virus of rabies. It therefore became necessary to try new and independent methods; for instance, cultivation of the rabies virus in glass.

Jenner was the first to propound the idea that the poison which used to be called " grease " in horses, but which we now more accurately describe as " horse-pox," must be attenuated in its poisonous activity, if I may use the expression, by being transmitted through cows before it could be introduced without danger into the system of man. This induced us to think it might be possible to attenuate the rabies virus by passing it through the bodies of certain animals. Many attempts were made, but in the majority of the experiments on animals the poison increased in virulence, just as in rabbits and Guinea-pigs; fortunately this was not so in the case of monkeys.

On December 6, 1883, the bulb of a dog, which was known to be mad from the fact that a child which it had bitten had died of hydrophobia, was used to inoculate a monkey by trephining. The monkey was attacked with rabies eleven days later; from the first monkey the virus was transmitted to a second one, which was also attacked with rabies after eleven days' interval. In a third monkey rabies declared itself after an interval of twenty-three days, and so on. With the bulb of each of these monkeys two rabbits were inoculated by trephining. The rabbits which were inoculated from the first monkey were seized with rabies after intervals of thirteen and sixteen days respectively; those inoculated from the second monkey after fourteen and twenty days; those from the third after twenty-six and thirty days; those from the fourth after twenty-eight days in each case; those from the fifth after twenty-seven days, and those from the sixth after thirty days.

It is thus impossible to doubt that by transmission from monkey to monkey, and from the different monkeys to rabbits, the strength of the poison is weakened in the latter just as it is weakened in the dog. A dog which was inoculated with the bulb of the fifth monkey had an incubation period of not less than fifty-eight days, although inoculation was performed by trephining. Other experiments of the same nature, which were performed on a series of monkeys, led to results of a like character. We are thus in possession of a method which enables us to attenuate the virulence of rabies. Successive transmissions from monkey to monkey produce a virus which, on being transmitted to rabbits, communicates rabies after an incubation period, the length of which gradually increases. If, on the other hand, one passes on from these rabbits to successively inoculate fresh rabbits, the rabies come under the law of increasing virulence on transmission from rabbit to rabbit, of which I have already spoken. The application of these facts yields a method of vaccinating dogs as a protection against rabies We take as a starting point one of the rabbits which have been inoculated from monkeys to such a sufficient degree that hypodermic or intravenous injection does not cause death. The succeeding preventive inoculations are performed with the virus-containing bulbs of the rabbits which have been the subjects of successive transmissions of infection from rabbit to rabbit, proceeding from the first infected.

In our experiments we have as a rule employed inoculation with virus from rabbits which have died after an incubation period of four weeks, but three or four times we have renewed our preventive inoculation from the bulbs of rabbits which have been inoculated from the rabbit which served as our point of departure.

After I had brought into use this method of vaccinating dogs as a protection against rabies, and had collected a large number of dogs which were rendered insusceptible of the disease, foreseeing a more extensive application of the method, and remembering the opposition which was at first shown to Jenner's discovery, I determined t lay before a scientific commission such of my results as it wa obvious must serve in the future as the basis for the vaccination of dogs for rabies.

The Under Minister of Instruction, M. Tallierese, to whom I mentioned my project, was willing to support it, and appointed MM. Beclard, Paul Bert, Bouley, Tisserand, Villemin and Vulpian to inquire into the facts, which I had already communicated to the Academy of Sciences at its meeting on May 29th. After having chosen M. Bouley as President, and M. Villemin as Secretary, the commission at once set to work, and I have the satisfaction of being able to tell you that it has quite recently presented its first report to the minister.

I will now give a brief account of the results with which the first report of this commission deals. I presented to the commission nineteen vaccinated dogs, all of which had been rendered insusceptible by preventive inoculation, and thirteen of which after vaccination had been proved by inoculation by trephining. These nineteen dogs were compared in different ways with nineteen dogs chosen from others for the purpose of the experiments. In the first place, on the 1st of June, two of the protected dogs and two of the trial dogs were inoculated by trephining under the dura mater with the bulb from a mad street-dog. On the 3d of June, one protected dog and one trial dog were bitten by a mad street-dog. On the 4th of June, the commission made the same mad dog bite ancther protected and another trial dog. On the 6th of June, the mad dog which had been used on the 3d and 4th of June died, and with its bulb three protected dogs and three trial dogs were inoculated by trephining. On the 10th of June, the commission had one protected dog and one trial dog bitten by a fresh mad dog from the streets. On the 16th of June, the commission had two fresh dogs, one protected and one trial dog, bitten by one of the trial dogs of the 1st of June, which had gone mad on the 14th, as a result of the trephining performed on the 1st of June. On the 19th of June, the commission had three protected and three trial dogs inoculated in one of the popliteal veins with the bulb of a mad street dog. On the 20th of June, the commission also had ten dogs—viz. six protected and four trial dogs, chosen from several others—inoculated in a vein. On the 28thof June, it having been brought to the knowledge of the commission that a veterinary surgeon, M. Paul Simon, had a mad dog in his hospital, four dogs were brought to it—viz., two protected and two trial dogs—in order that it might bite them.

The commission on rabies has thus performed experiments on thirty-eight dogs, nineteen of which had been supplied by me as insusceptible to rabies, while the other nineteen could be made mad. Those of the dogs which have not died as a result of the experiments are under observation, and will be kept under it for a long time. As to the present condition of the dogs which have been the subject of inquiry, the commission report that, in the case of the nineteen trial dogs, of six which were bitten rabies occurred in three; of seven which were inoculated in a vein it occurred in five, and of five which were inoculated by trephining it occurred in all, while *not a single sign of rabies has shown itself in any of the nineteen vaccinated dogs.*

During the course of the inquiry one of the protected dogs died on the 13th of July from a sanguineous diarrhœa, which first declared itself in the early days of that month. In order to determine whether rabies had any share in its death, three rabbits and one Guinea-pig were at once inoculated with its bulb by trephining. All of these four animals are still in the best of health, which is a certain proof that the dog did not die of rabies, but of a common disease.

The next report of the commission will contain information as to the insusceptibility to rabies of twenty dogs which have been vaccinated by the commission itself.—*N. Y. Med. Journal.*

